# The Impact of Pneumoperitoneum on Mean Expiratory Flow Rate: Observational Insights from Patients with Healthy Lungs

**DOI:** 10.3390/diagnostics14212375

**Published:** 2024-10-24

**Authors:** Lajos Bogár, Kamilla Domokos, Csaba Csontos, Balázs Sütő

**Affiliations:** Department of Anaesthesia and Intensive Care, Medical School, University of Pécs, 7624 Pécs, Hungary; bogar.lajos@pte.hu (L.B.);

**Keywords:** laparoscopic surgery, pneumoperitoneum, mechanical ventilation, chest compliance, expiratory flow rate, elastance, airway resistance, inspiratory-to-expiratory ratio, body mass index

## Abstract

Background/Objectives: Surgical pneumoperitoneum (PP) significantly impacts volume-controlled ventilation, characterized by reduced respiratory compliance, elevated peak inspiratory pressure, and an accelerated expiratory phase due to an earlier onset of the airway pressure gradient. We hypothesized that this would shorten expiratory time, potentially increasing expiratory flow rate compared to pneumoperitoneum conditions. Calculations were performed to establish correlations between respiratory parameters and the mean increase in expiratory flow rate relative to baseline. Methods: Mechanical ventilation parameters were recorded for 67 patients both pre- and post-PP. Ventilator settings were standardized with a tidal volume of 6 mL/kg, a respiratory rate of 12 breaths per minute, a PEEP of 3 cmH_2_O, an inspiratory time of 2 s, and an inspiratory-to-expiratory ratio of 1:1.5 (I:E). Results: The application of PP increased both peak inspiratory pressure and mean expiratory flow rate by 28% compared to baseline levels. The elevated intra-abdominal pressure of 20 cmH_2_O resulted in a 34% reduction in dynamic chest compliance, a 50% increase in elastance, and a 20% increase in airway resistance. The mean expiratory flow rate increments relative to baseline showed a significant negative correlation with elastance (*p* = 0.0119) and a positive correlation with dynamic compliance (*p* = 0.0028) and resistance (*p* = 0.0240). Conclusions: A PP of 20 cmH_2_O resulted in an increase in the mean expiratory flow rate in the conventional I:E ratio in the volume-ventilated mode. PP reduces lung and chest wall compliance by elevating the diaphragm, compressing the thoracic cavity, and increasing airway pressures. Consequently, the lungs and chest wall stiffen, requiring greater ventilatory effort and accelerating expiratory flow due to increased airway resistance and altered pulmonary mechanics. Prolonging the inspiratory phase through I:E ratio adjustment helps maintain peak inspiratory pressures closer to baseline levels, and this method enhances the safety and efficacy of mechanical ventilation in maintaining optimal respiratory function during laparoscopic surgery.

## 1. Introduction

Laparoscopic surgery is now essential in modern operating rooms, offering many patient benefits. However, requirements like extreme positioning and pneumoperitoneum can impact physiology, potentially causing complications not seen in open procedures and can be difficult to manage [1]. It provides numerous benefits compared to open procedures, including minimized trauma, faster recovery, reduced pain, and shorter hospital stays [2,3,4,5]. In abdominal laparoscopic surgery, the insufflation of carbon dioxide (CO_2_) into the abdominal cavity is a critical step that creates pneumoperitoneum (PP), allowing for improved visualization and access to the surgical site; however, the elevated pressure distends the abdominal wall and other structures, creating a more rigid operating field [6]. Different factors, such as demographic and anatomical characteristics, influence abdominal compliance, which represents the slope of the pressure–volume (P–V) curve of the abdominal cavity and is a measure of the ease of abdominal dilatation [7]. This procedure induces significant wall tension due to the increased intra-abdominal pressure. The insufflation of CO_2_ restricts the movement of the diaphragm, the primary muscle involved in respiration. This restriction occurs because the diaphragm is displaced upwards by the pressure caused by the insufflated gas. The upward displacement reduces the thoracic cavity volume, leading to decreased lung expansion and potential respiratory compromise. As a result, the ventilation parameters of the lungs are also affected. In the pressure-controlled mode, maintaining a consistent peak inspiratory pressure often results in a decreased tidal volume (TV). Conversely, in the volume-controlled mode, the TV remains constant, but the peak inspiratory pressure can increase significantly. These physiological changes necessitate careful intraoperative monitoring and management to ensure patient safety and optimize surgical outcomes in order to prevent postoperative pulmonary complications [8,9]. According to the literature and previous studies, these changes in ventilation parameters are primarily attributed to a reduction in chest compliance [10,11]. The diminished compliance, resulting from increased abdominal pressure, significantly impacts respiratory mechanics during laparoscopic procedures. The application of intra-abdominal pressure ranging from 12 to 15 mmHg (16–20 cmH_2_O) is essential for achieving adequate surgical access. However, this pressure level has several physiological implications, diminishing the descent of the diaphragm during respiratory cycles and resulting in a decline in lung compliance [12]. Moreover, the increased intra-abdominal pressure leads to elevated peak inspiratory pressure and a reduction in functional residual capacity [13]. PP, while providing the necessary exposure for surgery, also introduces challenges to circulatory dynamics [14]. It interferes with both arterial and venous circulation, potentially contributing to the formation of atelectasis and causing ventilation/perfusion imbalances [15,16]. As a consequence, there may be a subsequent decrease in the partial pressure of oxygen in the arterial blood. Clinicians should be aware of these effects and carefully manage patient care and intra-abdominal pressure during surgical procedures to minimize respiratory and circulatory complications and ensure optimal patient outcomes [17,18,19].

Based on the literature review, several research groups have already suggested that after completion of PP, the previous conventional 1:2 ratio of inspiration to expiration time can be modified and shifted to a 1:1 ratio, or even to the reverse ratio of 2:1 in order to shorten the expiratory time and reduce the inspiratory peak pressure, improving oxygenation and enhancing ventilation [20,21,22,23,24,25]. However, it has not yet been thoroughly investigated which factors facilitate and enable this possibility of inverse proportionality.

Our research team noticed a reduction in expiration time in the volume-controlled mode displayed on the anesthesia machine screen after introducing PP, as observed in some previous research [19,26]. Since most respirators do not directly indicate the length of expiratory time and the average expiratory flow rate, we decided to investigate whether PP really accelerates expiratory flow rate and, thus, shortens expiratory time. Furthermore, we also intended to investigate which ventilation parameters these changes can be correlated with, clarifying the factors contributing to the acceleration of expiration.

This prospective observational descriptive study aims to uncover the physiological mechanisms behind alterations in respiratory dynamics caused by PP, offering valuable insights that may aid in refining ventilation strategies in the context of laparoscopic procedures. The inclusion of these patients targeted to evaluate the impact of PP on mechanical ventilation parameters, providing a thorough examination of respiratory dynamics under controlled intra-abdominal pressure conditions.

## 2. Materials and Methods

### 2.1. Patients

A cohort of 67 consecutive patients from the Department of Surgery at the Medical School, University of Pécs, scheduled for elective laparoscopic abdominal surgery, were prospectively enrolled in this observational, descriptive research. The distribution of patients in terms of indications is as follows: cholecystectomy (*n* = 47), intra-abdominal tumors (colon *n* = 7, gastric *n* = 2, suprarenal adenoma *n* = 2), and other procedures included (appendectomy *n* = 2, inguinal hernia repair *n* = 5, gastric banding surgery *n* = 1, and Tenckhoff catheter insertion *n* = 1). All participants provided written informed consent prior to their inclusion within the study. Exclusion criteria encompassed individuals with chronic obstructive pulmonary disease (COPD), bronchial asthma, silicosis, and a history of previous lung surgery. Additionally, patients under 18 years of age, those who did not provide informed consent, and pregnant individuals were excluded from the study. The rigorous selection process ensured a homogeneous study population, minimizing potential confounding factors related to pre-existing pulmonary conditions, developmental considerations, and gestational changes that could impact the study outcomes. The prospective, observational study received approval from the Regional Ethics Committee of the Medical School, University of Pécs, Hungary, and was conducted in strict accordance with the principles outlined in the Declaration of Helsinki. The study protocol was assigned permission number 9746—PTE—2023, granted on 3 November 2023, ensuring adherence to ethical standards and guidelines for research involving human subjects. The investigation commenced on 8 November 2023 and concluded on 31 January 2024. The data collection was formally registered with the Main Board of the Medical School, University of Pécs, ensuring compliance with institutional standards, and recorded at www.clinicaltrials.gov under the identifier NCT06212258 on January 2024—this registration serves to enhance transparency and accountability in accordance with international clinical trial guidelines.

### 2.2. Anesthesia and Respiratory Parameters

As standard practice, patients received oral Alprazolam (EGIS, Budapest, Hungary) premedication at doses of 0.25–0.50 mg in the evening preceding the operation. Comprehensive monitoring, including ECG, non-invasive blood pressure monitoring, and pulse oximetry (Infinity Delta monitor, Dräger Medical, Lübeck, Germany), was consistently employed for all patients throughout the procedure. Patients underwent pre-oxygenation with an inspired oxygen fraction (FiO_2_) ranging from 0.8 to 0.9 to optimize oxygen reserves prior to induction of general anesthesia. This was followed by the intravenous administration of 1.5 µg kg^−1^ of fentanyl (Kalceks, Riga, Latvia) and 2.0 to 2.5 mg kg^−1^ of propofol (Fresenius Kabi, Badhomburg, Germany) to achieve smooth and controlled anesthesia induction. Atracurium (Aspen Pharma, Dublin, Ireland) was then administered at a dose of 0.5 mg kg^−1^ to ensure effective neuromuscular blockade, facilitating safe endotracheal intubation under controlled conditions. Throughout the surgical procedure, anesthesia was maintained with sevoflurane (AbbVie, Rome, Italy), carefully adjusted to achieve a targeted minimal alveolar concentration (MAC) level between 0.9 and 1.1. The sevoflurane was vaporized into the fresh gas flow at a rate of 1.0 L min^−1^, with the medical air enriched to 60% oxygen concentration, ensuring optimal respiratory support and depth of anesthesia. Following anesthesia induction, patients’ lungs were ventilated using a volume-controlled mode (Perseus A500 ventilator, Dräger Medical, Lübeck, Germany). Ventilatory parameters included a TV set at 6 mL per kg^−1^ of body weight, a respiratory rate of 12 breaths per minute, a PEEP of 3 cmH_2_O, and an inspiratory time of 2 s. The data acquisition process involved positioning patients in a supine and horizontal orientation, ensuring standardized positioning conducive to accurate monitoring and data collection throughout the course of the investigation. Prior to the initiation of PP, baseline measurements (“before”) were meticulously recorded. Subsequently, “after” values were obtained approximately 3 to 4 min following the completion of PP using the High Flow Insufflation Unit (UHI-4, Olympus Medical Systems Corp., Tokyo, Japan), coinciding with the establishment of intra-abdominal pressure at 20 cmH_2_O. This systematic approach ensured accurate evaluation of immediate physiological reactions to increased intra-abdominal pressure during the surgical intervention. The working pressure was set to 16 cmH_2_O for the remainder of the surgical procedure. The respiratory settings were kept consistent during the two recorded time points. The respiratory parameters were calculated and recorded using the integrated factory software of the anesthesia machine, ensuring standardized and reliable data acquisition throughout the study. Our focus was exclusively on the immediate effects of PP during the first 30 min.

### 2.3. Statistical Analysis

Statistical analyses were performed using the Statistical Package for the Social Sciences (IBM, SPSS, version 24.0), and a *p*-value of less than 0.05 was considered statistically significant. Power analysis was performed, with a significance level (α) of 0.05 and power (β) of 0.20, which determined that a minimum of 65 patients was necessary for adequate statistical power. The distribution of the data was tested using the Kolmogorov–Smirnov test, which indicated that our data did not follow a normal distribution. Data are reported as median values with interquartile ranges. Statistical significance was assessed using the Wilcoxon rank sum test, and Pearson correlation coefficients were calculated to analyze statistical relationships among the parameters of interest. These methodologies were selected to ensure proper statistical analysis and accurate interpretation of the study results.

## 3. Results

Demographic data for the 67 patients included in the study are presented in Table 1. As a consequence of PP, both peak inspiratory pressure and expiratory flow rate exhibited a notable increase of 28%. The elevation of intra-abdominal pressure to 20 cmH_2_O resulted in a significant reduction in dynamic chest compliance by 34%, along with increases in elastance by 50% and airway resistance by 20% (all *p* < 0.0001, Table 2). Upon completion of PP, the fraction of inspired oxygen (FiO_2_) and the partial pressure of end-tidal carbon dioxide (PetCO_2_) experienced reductions of 3.3% and 3.0%, respectively. Despite the application of PP, TV remained unchanged due to the use of volume-controlled ventilation. However, a statistically significant difference of less than half percent was observed, which, while statistically significant, was deemed clinically irrelevant based on the findings presented in Table 2.

**Figure 1:**
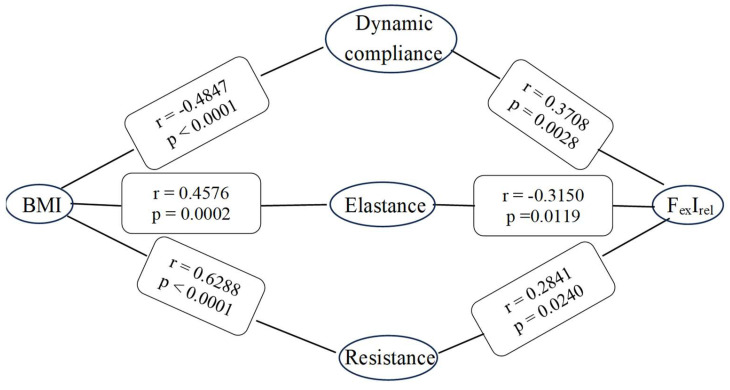
Pearson correlation coefficients were calculated to assess the relationships between body mass index (BMI) and the respiratory parameters: dynamic compliance, elastance, and resistance. Additionally, the correlations between the relative increase in mean expiratory flow rate from baseline (FexIrel) and the changes in dynamic compliance, elastance, and resistance resulting from the induction of pneumoperitoneum were evaluated.

Prior to PP application, a notable finding in our study revealed a significant negative correlation between body mass index (BMI) and dynamic chest compliance (r = −0.4847, *p* < 0.0001). Additionally, we observed a significant positive correlation between dynamic chest compliance and the relative increment in expiratory flow rate (r = 0.3708, *p* = 0.0028). The elastance values show similar correlations as the dynamic compliance, albeit with opposite signs, as the two parameters are reciprocals of each other. The highest and strongest correlation was observed between BMI and the resistance (*r* = 0.6288, *p* < 0.0001); however, it is noteworthy that the relative expiratory increment is only significantly correlated with the resistance (*r* = 0.2841, *p* = 0.0240); data are presented in Supplementary Table S1. The associations between body mass index (BMI) and the respiratory parameters—dynamic compliance, elastance, and resistance—are depicted in Figure 1 below. (Data are presented in section Supplementary Table S2).

## 4. Discussion

Our study demonstrated a significant rise in peak inspiratory pressure during volume-controlled ventilation when intra-abdominal pressure reached 20 cmH_2_O, highlighting the profound impact of increased intra-abdominal pressure on respiratory mechanics. Concurrently, we observed a reduction in both lung and chest wall dynamic compliance (34%), coupled with increase in elastance (50%) and rise in airway resistance (20%), indicative of pronounced alterations in respiratory mechanics likely attributable to elevated intra-abdominal pressure. The significant increases in peak inspiratory pressure and expiratory flow rate due to PP can be attributed to changes in airway pressure, which result from reduced lung and chest wall compliance caused by diaphragm and chest wall stiffening [27]. This observation highlights the profound impact that intra-abdominal pressure exerts on respiratory mechanics during laparoscopic surgery, emphasizing the critical need for continuous, vigilant monitoring and the meticulous adjustment of ventilatory parameters to optimize pulmonary function and mitigate the risk of perioperative respiratory complications. The results further delineate how pneumoperitoneum induces significant alterations in respiratory dynamics, including a marked reduction in chest wall compliance, increased lung stiffness, and elevated airway resistance, all of which necessitate careful management to ensure patient safety and favorable outcomes. Expiratory time decreased due primarily to the acceleration in average expiratory flow rate, reflecting expedited lung emptying. This acceleration indicates a faster release of air from the lungs, reflecting the altered respiratory dynamics caused by the conditions studied, particularly the increased intra-abdominal pressure [28]. Upon completion of PP, FiO_2_ decreased by 3.3% and PetCO_2_ by 3.0%, indicating significant shifts in respiratory dynamics and gas exchange efficiency due to elevated intra-abdominal pressure. The reduction in FiO_2_ may suggest a potential dilution effect or impaired oxygen delivery, while the decline in PetCO_2_ points to alterations in alveolar ventilation and ventilation/perfusion matching during PP. During PP insufflation, the hyperdynamic state or decreased cardiac output at higher abdominal pressures could potentially influence expired CO_2_ levels [29]. As we observed a significant positive correlation between dynamic chest compliance and the relative increment in expiratory flow rate, this association underscores the importance of maintaining adequate chest compliance for optimizing respiratory function and efficiency during surgical interventions. These findings underscore the need for careful management of respiratory parameters to maintain optimal gas exchange and minimize the risk of adverse respiratory effects. An interesting pattern emerged in the magnitude of changes induced by PP, as airway resistance increased the least, by only 20% on average. Conversely, dynamic compliance deteriorated significantly, decreasing by 34% compared to baseline. These effects were largely counterbalanced by a 50% increase in elastance. Consequently, mean expiratory flow rate was accelerated by 28%, and expiratory time was shortened by 27%. PP leads to increased lung elastance by restricting diaphragm movement and also reducing lung volume due to increased intra-abdominal pressure, diaphragmatic restriction, and increased lung stiffness. These pathophysiological changes can lead to increased work of breathing, impaired gas exchange, and increased risk of atelectasis. Factors such as obesity, pulmonary comorbidities, and age can exacerbate the respiratory effects of pneumoperitoneum [10].

Extended inspiratory time in mechanical ventilation offers several advantages, provides additional time for effective gas exchange, potentially enhancing oxygenation levels, especially in patients with compromised lung function [24,30]. Prolonged inspiratory phases facilitate the efficient removal of CO_2_ from the lungs, thereby aiding in the management of respiratory acidosis; longer inspiratory durations help in reducing peak airway pressures, thereby lowering the risk of barotrauma or volutrauma, particularly crucial for patients with delicate lung tissue. Additionally, extended inspiratory times can assist in the recruitment of collapsed alveoli, improving overall lung compliance and optimizing ventilation/perfusion matching in the pulmonary system [31,32]. These advantages underscore the critical role of prolonged inspiratory times in optimizing respiratory support and potentially improving patient outcomes in critically ill patients not only during anesthesia but also in intensive care settings. However, there are several disadvantages associated with longer inspiratory times during mechanical ventilation [24,33]. Excessive inspiratory durations can lead to inadequate ventilation, resulting in hypoventilation and potentially causing hypercapnia, particularly in patients with high CO_2_ production or reduced respiratory drive. Prolonged inspiratory phases may contribute to the development of intrinsic positive end-expiratory pressure (auto-PEEP), which can impair cardiac output and lead to hemodynamic instability. Longer inspiratory times may increase the risk of patient–ventilator asynchrony, potentially causing discomfort, ineffective ventilation, and increasing the likelihood of lung injury [34]. Extended inspiratory durations can also elevate mean airway pressures, thereby increasing the risk of barotrauma, such as pneumothorax, or volutrauma, such as ventilator-induced lung injury [35]. Prolonged inspiratory phases may delay the initiation of expiration, leading to insufficient exhalation time and potentially resulting in air trapping within the lungs. These disadvantages highlight the importance of carefully monitoring and adjusting inspiratory times to optimize patient ventilation and minimize potential complications during mechanical ventilation in clinical practice [36,37].

The post-expiratory pause, often employed during mechanical ventilation, may offer several potential benefits in clinical practice. This pause allows for the complete exhalation of gasses from the lungs, facilitating adequate gas exchange and preventing air trapping. By extending the expiratory phase, it can help reduce intrinsic positive end-expiratory pressure, which is beneficial in patients with compromised lung compliance or those at risk of ventilator-induced lung injury. Additionally, the post-expiratory pause allows for better synchronization between the patient’s respiratory efforts and the ventilator, potentially reducing the risk of patient–ventilator asynchrony. Furthermore, this pause provides a brief period of rest for respiratory muscles, which can be particularly advantageous in patients with respiratory fatigue or acute respiratory distress syndrome (ARDS) [38,39]. The duration of the post-expiratory pause has to be carefully adjusted based on individual patient needs and respiratory mechanics to optimize ventilation and improve patient outcomes during mechanical ventilation [40].

The significant positive correlation observed between dynamic chest compliance and the relative increment in expiratory flow rate indicates that improved chest compliance is linked to enhanced expiratory airflow dynamics. This association is likely attributed to better lung mechanics (elasticity, muscle activity) and reduced airway resistance. Patients with higher dynamic chest compliance generally experience greater enhancements in expiratory flow rates during breathing cycles [41]. As a consequence of PP, we observed a decrease in dynamic compliance and an increase in airway resistance. Modifying the I:E ratio could potentially improve respiratory mechanics.

The effect of an equal or inverted inspiratory-to-expiratory ratio (I:E) on peak inspiratory pressure (Ppeak), pressure expiratory gradient, and expiratory flow is significant in the context of PP and mechanical ventilation. In case of an equal ratio (1:1), the inspiratory phase duration is increased compared to the conventional 1:2 ratio, which can distribute the inspiratory pressure more evenly and may lower Ppeak. However, Ppeak might not change significantly if the total ventilation volume remains constant. The pressure gradient between inspiration and expiration becomes more balanced, potentially improving gas exchange efficiency by allowing longer inspiration times and maintaining more consistent alveolar pressures. There is generally sufficient time for expiratory flow, potentially reducing the risk of dynamic hyperinflation and air trapping. However, in patients with obstructive lung disease, this might still be insufficient if they require a longer expiratory phase. An inverted ratio (2:1), which extends the inspiratory phase longer than the expiratory phase, can increase Ppeak due to the prolonged application of pressure during inspiration. While this can be beneficial for improving oxygenation, it might also increase the risk of barotrauma. A shorter expiratory phase can result in a higher pressure gradient since there is less time for pressure to drop during expiration. While this might enhance oxygenation, it can also increase the risk of incomplete expiration, leading to air trapping and elevated end-expiratory pressure. A shorter expiratory phase can lead to higher initial expiratory flow rates but may not provide enough time for complete exhalation, especially in patients with increased airway resistance. This can result in air trapping, increased functional residual capacity, and potentially higher intrinsic PEEP [42]. The shorter expiration time makes it possible to set the inspiration time to be longer and thereby approach the implementation of the principles of lung protective ventilation. This method, namely the 1:2 ratio of I:E time, has been proposed by others to be changed to 1:1 or even to 2:1 [11,23,24,25]. Utilizing 1:1 or 2:1 ratio might improve respiratory mechanics and arterial gas exchanges [23,24,43]. However, previous research has shown that equal ratio ventilation did not improve respiratory mechanics or cerebral perfusion pressure during laparoscopy in the Trendelenburg position [23]. It is important to note that the exhalation time of 3 s, which remained after the 2 s inhalation of the 5 s breathing cycle, was sufficient for all our patients to maintain adequate oxygen saturation. An equal ratio (1:1) may help balance I:E pressures and flows, potentially improving gas exchange and reducing peak pressures, while an inverted ratio (2:1) may enhance oxygenation by prolonging inspiration, but it increases peak pressures and the risks of air trapping and barotrauma. A longer inspiratory time aimed at reducing Ppeak could eventually decrease the pressure gradient and expiratory flow.

The driving pressure “Pplat-PEEP” is the parameter primarily influencing the pressure gradient, which allows increased expiratory mean flow and reduced expiratory time during mechanical ventilation, and it is the pressure needed for alveolar opening. It represents the pressure gradient driving gas flow, directly impacting TV and oxygenation. Driving pressure depends on PEEP and TV. Consequently, adjusting PEEP and TV can potentially reduce driving pressure. Driving pressure-guided ventilation could be an effective method to reduce postoperative pulmonary complications and enhance recovery in patients undergoing laparoscopic surgery [44,45].

Correlation analysis revealed significant associations between dynamic compliance, elastance, and resistance with both BMI and the relative increase in mean expiratory flow rate from baseline. Obesity appears to exert a systematic restrictive effect on the chest wall, particularly affecting the ribcage rather than the lungs, especially when the individual is in a supine position [46]. These findings suggest that BMI and changes in expiratory flow rate are important factors influencing respiratory mechanics under the conditions studied [47]. The reduced functional residual capacity (FRC) and expiratory reserve volume, along with a high closing volume to FRC ratio in obesity, are linked to the closure of peripheral lung units, abnormalities in the ventilation/perfusion ratio, and hypoxemia, particularly when in the supine position. Prior to PP application, we found a significant negative correlation between BMI and dynamic chest compliance. This implies that higher BMI is associated with reduced dynamic chest compliance, indicating potential implications for respiratory mechanics in obese patients undergoing laparoscopic procedures. Obesity can lead to notable changes in the respiratory system, as studies have demonstrated a marked exponential decrease in respiratory system compliance with increasing body mass index [48,49,50]. From assessing lung function in morbidly obese individuals, the vital capacity (VC) was found to be reduced to 75% of the predicted value [51]. The result emphasizes the substantial impact of BMI on resistance while highlighting a specific association between relative expiratory increment and resistance in our analysis.

In clinical settings, the choice of inspiratory time should be tailored to individual patient factors such as lung function, respiratory effort, and overall medical condition. It is crucial to closely monitor and adjust ventilator settings to enhance gas exchange efficiency and mitigate the risks associated with prolonged inspiratory durations. It is imperative for future research to explore how the application of PP affects ventilation mechanics in patients with COPD. This area warrants thorough investigation to deepen our understanding of the complex dynamics involved. Such research could reveal important implications for individuals with pre-existing respiratory conditions, leading to improved management strategies and more tailored interventions to meet their specific needs. No adverse events related to the observations were noted. There was no indication that subjects with a greater increase in airway resistance or elastance or a greater decrease in dynamic compliance required a longer duration of ventilatory support post-surgery. The study did not identify any postoperative complications directly linked to these respiratory changes.

Our study has some important limitations. Notably, our research team did not independently verify the accuracy of the compliance, elastance, and resistance values calculated by the anesthesia machine. Future research should involve a comprehensive validation of these measurements to confirm the reliability and accuracy of the data. This process is essential for bolstering the integrity of the findings and increasing the credibility of the study’s conclusions. Although patients’ height and weight were measured as part of the preoperative assessment a few days before surgery, the data used on the day of the procedure were based on self-reported information. Esophageal pressure measurements and assessments of preoperative respiratory function, including parameters such as compliance, elastance, and resistance, were not conducted in this study. Additionally, severe smoking, which can significantly impact respiratory mechanics, was not accounted for. This omission underscores the need for future studies to incorporate these assessments to provide a more comprehensive understanding of baseline respiratory characteristics. Such data would offer valuable insights into how these variables affect responses to pneumoperitoneum and overall perioperative respiratory dynamics. For calculating TV, we opted to use total body weight instead of ideal body weight. This approach was chosen to maintain physiological partial pressure of end-tidal carbon dioxide, particularly in obese patients, throughout the intraoperative period [52]. The median BMI of the study population was higher than the ideal BMI. Respiratory mechanics differ in obese patients compared to non-obese patients (the respiratory pressures are higher due to the increased weight of the chest wall, and the functional capacity is lower). Therefore, the results cannot be entirely extended to the whole population.

### Clinical Implications

The reduction in expiratory time, accomplished by minimizing the post-expiratory pause, enables the adjustment of the conventional I:E ratio from the standard 1:2 to alternative configurations such as 1:1 or even 2:1. This modification allows for prolonged inspiratory phases, potentially improving oxygenation and gas exchange during mechanical ventilation in specific clinical scenarios. Extending the inspiratory time has the potential to reduce both peak and mean inspiratory pressures in the volume-controlled ventilation mode. By lowering the inspiratory pressures, it minimizes the risk of barotrauma and volutrauma, thereby reducing the likelihood of intraoperative and postoperative pulmonary complications. This approach enhances the safety and efficacy of mechanical ventilation in maintaining optimal respiratory function, offers significant advantages for lung-protective ventilation strategies during anesthesia for laparoscopic surgery. However, managing risks like air trapping, barotrauma, and patient–ventilator asynchrony is also crucial in clinical practice as they significantly affect patient safety and outcomes. Balancing intervention benefits with potential complications ensures better patient care and minimizes harm. In the future, we plan to expand our research by applying more refined selection criteria and conducting sub-group analyses.

## 5. Conclusions

In laparoscopic surgery, PP hinders the downward movement of the diaphragm while promoting its upward movement during volume-controlled respiration. As a result, higher peak inspiratory pressures are needed to maintain the same TV as before PP was induced. Moreover, expiration is faster compared to non-pressurized abdominal conditions. By adjusting the inspiratory-to-expiratory ratio (1:1, 2:1) to compensate for the reduced expiratory time, the inspiratory phase can be prolonged—dynamic respiratory parameters will be improving—helping to maintain peak inspiratory pressures closer to pre-PP levels. Employing lung-protective ventilation strategies during laparoscopic procedures can reduce the risk of intraoperative and postoperative pulmonary complications both in patients with healthy lungs and in patients with co-morbidities.

## Figures and Tables

**Table 1:** Patient characteristics.

Number of Patients	67
Male/female (*n*)	30/37
Operation type (*n*)	
LC	47
Intra-abdominal tumor (colon: 7, gastric: 2, suprarenal: 2)	11
Other (appendectomy: 2, inguinal hernia repair 5, gastric banding surgery: 1, and Tenckhoff catheter insertion: 1)	9
Age (years)	59 (49, 69)
Weight (kg)	81 (69, 93)
Height (cm)	170 (163, 175)
BMI (kg m^−2^)	28 (24, 31)

Data are given as median [interquartile values]. LC = laparoscopic cholecystectomy, BMI = body mass index.

**Table 2:** Ventilatory parameters measured immediately before and after application of pneumoperitoneum (*n* = 67).

Variable	Baseline	After PP	Change (%)	*p* Value
FiO_2_ (%)	61 (56, 61)	59 (50, 61)	−3.3	<0.0001
PetCO_2_ (mm Hg)	33 (30, 36)	32 (31, 35)	−3.0	0.0148
Tidal volume (mL)	459 (401, 539)	461 (388, 530)	+0.4	<0.0001
Peak inspiratory pressure (mbar)	14 (11, 18)	18 (15, 22)	+28	<0.0001
Expiratory time (s)	2.6 (2.2, 2.8)	1.9 (1.7, 2.2)	−27	<0.0001
Mean expiratory flow rate (mL s^−1^)	178 (151, 218)	228 (187, 281)	+28	<0.0001
Dynamic compliance (mL mbar^−1^)	56 (43, 69)	37 (31, 46)	−34	<0.0001
Elastance (mbar mL^−1^)	18 (14, 21)	27 (22, 30)	+50	<0.0001
Airway resistance (mbar L^−1^ s^−1^)	10 (8, 12)	12 (10, 14)	+20	<0.0001

Data are given as median [interquartile values]. PP = pneumoperitoneum, FiO_2_ = fraction of inspired oxygen, PetCO_2_ = partial pressure of end-tidal carbon dioxide.

## Data Availability

All data created or analyzed during this study are available from the corresponding author upon request.

## Supplementary Materials

The following supporting information can be downloaded at: https://www.mdpi.com/article/10.3390/diagnostics14212375/s1, Table S1: Pearson correlations coefficient between body mass index and dynamic compliance, elastance and airway resistance; mean expiratory flow rate increment relative to baseline and dynamic compliance, elastance and resistance due to application of pneumoperitoneum; Table S2: Pearson correlation coefficients between body mass index (BMI) baseline peak inspiratory pres-sure (PIP), mean expiratory flow rate increment relative to baseline (FexIrel), baseline dynamic compliance (Cdyn), baseline airway resistance (R), and baseline elastance (E).

## Glossary

PP = pneumoperitoneum; TV = tidal volume; PEEP = positive end-expiratory pressure; I:E = inspiratory-to-expiratory ratio; cmH_2_O = water pressure; mmHg = millimeter of mercury; CO_2_ = carbon dioxide; COPD = chronic obstructive pulmonary disease; BMI = body mass index; MAC = minimal alveolar concentration; FexIrel = mean expiratory flow rate increment relative to baseline; FRC = functional residual capacity
