# Supplementary material for: The Impact of Pneumoperitoneum on Mean Expiratory Flow Rate: Observational Insights from Patients with Healthy Lungs
Source: Diagnostics (Basel). 2024 Oct 24;14(21):2375. doi: 10.3390/diagnostics14212375 (PMC11544817; doi:10.3390/diagnostics14212375)
Supplement: Supplementary file 1 [file diagnostics-14-02375-s001.zip › Table S1.pdf]

Table S1. Pearson correlations coefficient between body mass index and dynamic compliance, elas-tance and airway resistance; mean expiratory flow rate increment relative to baseline and dynamic compliance, elastance and resistance due to application of pneumoperitoneum

	BMI	Dynamic compliance	FexIrel
r	-0.4847		0.3708
P value	< 0.0001		0.0028
	BMI	Elastance	FexIrel
r	0.4576		-0.3150
P value	0.0002		0.0119
	BMI	Resistance	FexIrel
r	0.6288		0.2841
P value	< 0.0001		0.0240

r = Pearson correlation coefficient, BMI = body mass index, FexIrel = mean expiratory flow rate increment relative to baseline.
