# Supplementary material for: The Impact of Pneumoperitoneum on Mean Expiratory Flow Rate: Observational Insights from Patients with Healthy Lungs
Source: Diagnostics (Basel). 2024 Oct 24;14(21):2375. doi: 10.3390/diagnostics14212375 (PMC11544817; doi:10.3390/diagnostics14212375)
Supplement: Supplementary file 1 [file diagnostics-14-02375-s001.zip › Table S2.pdf]

Table S2. Pearson correlation coefficients between body mass index (BMI) baseline peak inspiratory pressure (PIP), mean expiratory flow rate increment relative to baseline (FexIrel), baseline dynamic compliance (Cdyn), baseline airway resistance (R), and baseline elastance (E).

		BMI	PIP	FexIrel	Cdyn	R	E
BMI							
PIP	r	0.8213					
	p	<0.0001					
FexIrel	r	0.3808	-0.3776				
	p	0.0028	0.0012				
Cdyn	r	-0,4847	0.7461	0.3714			
	p	<0.0001	<0.0001	0.0028			
R	r	0.6288	0.2844	0.2841	-0.6227		
	p	<0.0001	0.0240	0.0240	<0.0001		
E	r	0.4576	-0.3151	-0.3150	-0.8329	0.7048	
	p	0.0002	0.0119	0.0119	<0.0001	<0.0001	
